# Crim1 suppresses left ventricular hypertrophy

**DOI:** 10.3892/br.2019.1241

**Published:** 2019-09-16

**Authors:** Long Yang, Jionghong He, Guiling Xia, Jun Yang, Qian Tang, Yongyao Yang, Jiusheng Deng

Biomed Rep 10:343-350, 2019; DOI: 10.3892/br.2019.1214

Following the publication of this article, the authors have realized that the first fund number in their paper (i.e., for the grant they received from the Guizhou Provincial Science and Technology Fund) was written incorrectly. Instead of “[grant no. (2019) 1209], it should have been written as “[grant no. (2019) **1205**]” (correction highlighted in bold).

The authors apologize to the funders of their research project, and to the readership of the Journal for any inconvenience caused.

